# The Urge to Fight: Persistent Escalation by Alcohol and Role of NMDA Receptors in Mice

**DOI:** 10.3389/fnbeh.2018.00206

**Published:** 2018-09-13

**Authors:** Herbert E. Covington, Emily L. Newman, Steven Tran, Lena Walton, Walae Hayek, Michael Z. Leonard, Joseph F. DeBold, Klaus A. Miczek

**Affiliations:** ^1^Department of Psychology, Tufts University, Medford, MA, United States; ^2^Neuroscience, Sackler School of Biomedical Sciences, Tufts University, Boston, MA, United States; ^3^Pharmacology, Sackler School of Biomedical Sciences, Tufts University, Boston, MA, United States; ^4^Psychiatry, Sackler School of Biomedical Sciences, Tufts University, Boston, MA, United States

**Keywords:** alcohol, aggressive behavior, motivation, glutamate receptors, NMDA/AMPA, tolerance, sensitization, neuroplasticity

## Abstract

Alcohol drinking, in some individuals, culminates in pathologically aggressive and violent behaviors. Alcohol can escalate the urge to fight, despite causing disruptions in fighting performance. When orally administered under several dosing conditions the current study examined in a mouse model if repeated alcohol escalates the motivation to fight, the execution of fighting performance, or both. Specifically, seven daily administrations of alcohol (0, 1.8, or 2.2 g/kg) determined if changes in the motivation to initiate aggressive acts occur with, or without, shifts in the severity of fighting behavior. Responding under the control of a fixed interval (FI) schedule for aggression reinforcements across the initial daily sessions indicated the development of tolerance to alcohol’s sedative effect. By day 7, alcohol augmented FI response rates for aggression rewards. While alcohol escalated the motivation to fight, fighting performance remained suppressed across the entire 7 days. Augmented FI responding for aggression rewards in response to a low dose of alcohol (1.0 g/kg) proved to be persistent, as we observed sensitized rates of responding for more than a month after alcohol pretreatment. In addition, this sensitization of motivated aggression did not occur with a general enhancement of motor activity. Antagonism of NMDA or AMPA receptors with ketamine, dizocilpine, or NBQX during later challenges with alcohol were largely serenic without having any notable impact on the expression of alcohol-escalated rates of FI responding. The current dissociation of appetitive and performance measures indicates that discrete neural mechanisms controlling aggressive arousal can be distinctly sensitized by alcohol.

## Introduction

Alcohol-escalated violence inflicts serious harm and suffering on a global scale as documented over many decades (Pernanen, 1993; Bye and Rossow, 2010). More than half of violent criminal acts are associated with alcohol in the perpetrator or victim or both (Beck et al., 2014). In such cases, alcohol consumption prompts a motivational state that culminates in attempts to act violently, which is distinct from impaired and uncoordinated behavior during intoxication (Mayfield, 1976; Jaffe et al., 1988). Cognitive models attempt to explain alcohol-instigated aggression through processes such as fear reduction, cortical disinhibition, anticipation of expected outcomes, or selectively attending to provocative cues (i.e., alcohol-induced myopia) (Schmutte et al., 1979; Pernanen, 1993; Pihl et al., 1993; Sayette et al., 1993; Zhang et al., 2002; Giancola et al., 2011). Here, we focus on the motivation to engage in aggressive behavior when it is ostensibly rewarding and outcomes are predictable in a mouse model (Ginsberg and Allee, 1942; Fish et al., 2005). The currently selected experimental conditions aim to systematically dissect how alcohol over repeated exposures alters the appetitive and performance (i.e., consummatory) components of aggressive behavior (Miczek et al., 2015; Golden et al., 2017; Hashikawa et al., 2017).

The neural circuitry of appetitive and consummatory behaviors overlap considerably (Wise, 2013). Quantification of appetitive behaviors, particularly when maintained by fixed interval (FI) schedules, indicates the state of “arousal” immediately prior to reward receipt (Wenger and Dews, 1976). This method of schedule-controlled behavior allows for dissociating motivational processes that precede performance measures (Gonzalez and Goldberg, 1977). Several types of aggression can be highly arousing and represent evolutionarily conserved, natural rewards (Scott, 1958). Reactive “hot” acts of violence are often produced by repeated cycles of alcohol intoxication (*see* Beck et al., 2014 *for a review of clinical data*). The neural architecture supporting such maladaptive aggression remains unknown, but key epidemiological findings provide some insight into their origins, including the predictably high rate of reoccurrence and their progressive escalation in alcohol use disorders (Fergusson and Horwood, 2000; Coid et al., 2006). Preclinical data corroborate these trends, such that the proportion of alcohol-heightened aggressors in a sample increases with a history of intermittent voluntary drinking (Fish et al., 2002; Hwa et al., 2015). We hypothesize that repeated exposures to alcohol - in certain contexts and when *winning* a confrontation is expected – can potentially trigger an intense motivation to engage in future aggressive acts.

The dose-dependent biphasic modulation of aggressive performance (Miczek et al., 1992, 1998) is clearly characterized by lower alcohol doses, which reliably increase threats and attacks; yet, the *motivational* indices prior to fighting require more evaluation (Fish et al., 2008). In addition to its acute effects on behavior, repeated EtOH administrations increase the propensity for the later expression of an alcohol-heightened aggressive phenotype (Lessov et al., 2001; Fish et al., 2002; Didone et al., 2016). In line with a dopamine-dependent theory of behavioral plasticity, increases in synaptic strength, particularly excitatory synapses on dopamine (DA) neurons in the ventral tegmental area (VTA), occurs after an *in vivo* administration of alcohol, like DA-positive modulators (Saal et al., 2003). Interestingly, levels of operant responding that are reinforced by aggression require intact DA receptor activation in the ventral striatum (Couppis and Kennedy, 2008). Moreover, persistently augmented behavioral responses to alcohol rely on the activation of *N*-methyl-*d*-aspartate (NMDA) and alpha-amino-3-hydroxy-5-methyl-4-isoxazolepropionic acid (AMPA) receptors in the VTA (Phillips and Shen, 1996; Broadbent et al., 2003).

The current objective was to determine how alcohol, when orally administered under several dosing conditions increases both (1) the motivation to fight, and (2) the execution of fighting. We sought to confirm that lower acute doses of alcohol will increase the intensity of offensive aggression (e.g., escalated number of bites and threats), without affecting the motivation to engage in fighting. The impairing effects of higher alcohol doses were expected to reduce both anticipatory responding and performance measures (Fish et al., 2008). We hypothesized that during repeated exposures to alcohol, tolerance first develops to the sedative effects, and eventually sensitized responding emerges, which may serve as an index of motivation. We determined whether or not changes in the motivation to initiate aggressive acts occur with, or without, shifts in the intensity of fighting behavior (Newman et al., 2018). Responding under the control of the FI schedule of reinforcement is a highly sensitive measure of appetitive behavior, indicating that the pattern of responding (i.e., the “scallop”) during successive administrations of alcohol may therefore be attributed to underlying changes in incentive-motivation. Thus, our final hypothesis explored to what extent the activation of ionotropic glutamate receptors (iGluRs) is necessary for maintaining any lasting changes in aggressive reinforcement resulting from repeated oral administrations of alcohol.

## Materials and Methods

### Subjects

Eight-week-old male C57BL/6J mice (C57; Jackson Labs, Bar Harbor, ME, United States) were housed in polycarbonate cages (28 cm × 17 cm × 14 cm) lined with pine chip bedding. Food and water were available at all times. For Experiments 1–4, “resident” male mice (*n* = 80) were housed with a female of the same age and strain for at least 1 month to facilitate aggressive behaviors and avoid social isolation (Crawley et al., 1975; Miczek and O’Donnell, 1978). All pups were culled at 3 weeks of age. Female partners were removed following the display of consistent aggression by the resident male, at which point these aggressive resident males were singly housed for the remainder of the experiment. For Experiment 5, 8-week-old male C57 mice (*n* = 22) were singly housed in polycarbonate cages for the duration of the experiment.

Additional male C57 mice (8 weeks) were group-housed as “intruders” in large polycarbonate cages (46 cm × 24 cm × 15 cm), with unrestricted food and water available. These intruder mice were used for daily tests of aggression by residents for approximately 1 week, and then replaced with a new cohort of intruders. The vivarium was maintained at 21 ± 1°C, 30–40% humidity, and 12-h reverse light/dark cycles (lights on at 17:30 h). Experimental procedures were approved by the Tufts Institutional Animal Care and Use Committee following the *Guide for the Care and Use of Laboratory Animals* (National Research Council 2011).

### Procedures

#### Measurements of Aggression and Aggressive Motivation

**FIGURE 1:**
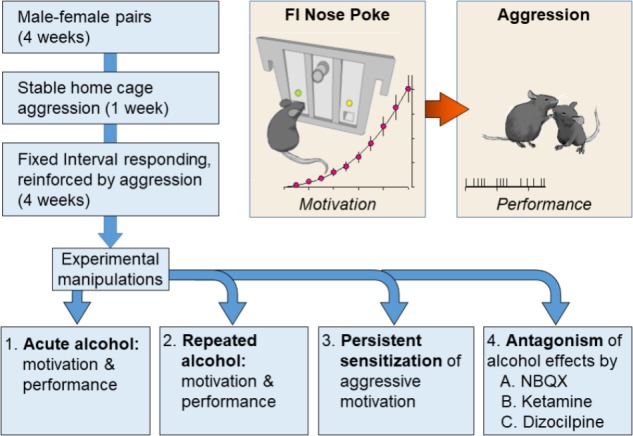
Resident male C57BL/6J (C57) mice were housed with breeding C57 females for at least 1 month. In daily resident–intruder confrontations, each resident male encountered a novel, male C57 intruder for 5 min in the resident home cage. After establishing an aggressive phenotype, each resident was trained during a fixed interval (FI) schedule that was reinforced by the presentation of an intruder. The FI was progressively increased from 1 s to 10 min over the course of 1 month. Mice were divided into experimental groups upon establishing consistent patterns of FI responding. *Experiment 1* revealed the effects of acutely administered water or EtOH (0.5, 1.0, or 1.8 g/kg, PO) on FI responding and subsequent aggressive behavior. *Experiment 2* evaluated the effects of repeated daily administrations of EtOH (1.8 or 2.2 g/kg, PO) on FI and aggression trials. *Experiment 3* confirmed and extended these findings by measuring the persistence of alcohol-escalated motivation to fight. *Experiment 4* examined the role of iGluRs during the expression of sensitized FI responding and aggressive performance in response to a 1.0 g/kg EtOH challenge that occurred at least 10 days after repeated administrations of water or EtOH (2.2 g/kg, PO).

After at least 4 weeks of cohabitation and the birth of one litter of pups to confirm successful mating behavior, each resident male was quantitatively screened for consistent display of aggressive behavior (See **Figure 1**). During this phase of screening for aggression, the female and pups were removed from the resident’s home cage, before an intruder was introduced (Miczek and O’Donnell, 1978). This social confrontation lasted for 5 min following an initial attack bite by the resident, and confrontations were permitted until a total of 30 bites accumulated, or 5 min elapsed if no fight occurred. An experimenter tallied the frequency of attack bites, and recorded the duration of the confrontation. Daily confrontations were conducted at 24-h intervals until each resident displayed a stable level of aggression toward an intruder (30 bites within 1 min, over seven sessions). By the final screening session >90% of residents were highly aggressive, displaying vigorous attacks (i.e., 30 bite limits reached within 1 min) with a short latency (<10 s) to the initial attack. Intruders were systematically rotated to ensure that the resident did not habituate to a specific intruder (Winslow and Miczek, 1983).

Once stable and reliable aggression was established, residents (*n* = 74, *six mice never established stable levels of aggression during screening trials*) were then conditioned to perform a nose poke task according to a FI schedule reinforced by the opportunity to fight (Fish et al., 2002). A panel with two nose-poke operanda was inserted into the resident’s home cage and affixed to the walls. The first nose poke in the assigned “active” hole after the interval had elapsed was reinforced by the presentation of an intruder which was promptly attacked. Specifically, after completing the behavioral requirement of the FI schedule, a house light was illuminated and an intruder was simultaneously introduced into the resident’s home cage. After 1 min of aggressive interactions, the intruder was removed, the house light turned off, and the session terminated. The response panel was removed from the resident’s home cage immediately after each daily session. During the first 2 weeks of conditioning, the female partner of the male resident was returned to the home cage at the completion of each FI session. After the first 2 weeks of FI training, the female was permanently removed and the male resident was singly housed under the same housing conditions. FI sessions were conducted daily for all experimental mice. On the first day of FI conditioning, the FI interval was 30 s. Over the next 30 daily FI sessions the interval was gradually increased to 10 min.

Once the FI reached 10 min, five to seven sessions were conducted per week until the mice demonstrated stable rates of responding in the “active” nose poke hole. Over successive daily sessions, the pattern of responses reliably increased in frequency toward the end of the FI (i.e., demonstrating an FI-typical “scalloped” pattern of responding). The scalloped pattern of nose pokes allows for assessments of the rate of responding and the index of curvature (Fry et al., 1960). This index of curvature ranges from a value of -0.75, indicating that all responses are made during the first quarter of the interval, to a value of +0.75, indicating that all responses are made during the last quarter of the interval. If responses are evenly distributed across the interval, the index of curvature is 0. Consistent with previous uses of this procedure the curvature values of all experimental mice for the current series of experiments approximated +0.30 (Fish et al., 2002).

#### **Experiment 1**: Effects of Acutely Administered Alcohol on Motivation to Fight and Fighting Performance

After stable rates of FI responding were observed (i.e., <20% variation in FI responding over 3 days), male residents (*n* = 10) were habituated to oral administrations of tap water via gavage (*per os*, PO) 10 min before each daily FI session for 1 week. After habituation to these handling procedures, residents were given either tap water or various doses of EtOH (0.5, 1.0, and 1.8 g/kg, PO) 10 min before FI sessions in an unsystematic sequence at 72 h intervals.

#### **Experiment 2:** Effect of Repeated Alcohol Administrations on the Motivation to Fight and Fighting Performance

After stable rates of FI responding were observed, male residents (*n* = 30) were habituated to oral administrations of tap water 10 min before each daily FI session for 1 week. These residents were given seven consecutive days of either water (*n =* 10) or one of two different doses of EtOH (1.8 or 2.2 g/kg/day, PO, *n* = 10/dose) 10 min before their daily FI session. Ten days after their last daily dose of water or EtOH, mice were orally administered 1.0 g/kg EtOH to assess the potentially sensitizing effects of repeated EtOH administration on FI responding and fighting.

#### **Experiment 3:** The Long-Term Consequences of Repeated EtOH on the Motivation to Fight and Fighting Performance

Once stable rates of FI responding were observed, male residents (*n* = 14) were habituated to oral administrations of tap water via gavage prior to each daily FI session. Each of these residents was then given EtOH (1.8 g/kg/day, PO) 10 min prior to each daily FI session for seven consecutive days, adhering to a within-subjects design. To carefully observe the long-term effects of these repeated, intermittent EtOH administrations on FI responding and fighting performance, these mice were subsequently challenged with EtOH (1.0 g/kg, PO) 14, 40, and 60 days after the last 1.8 g/kg dose. Five days prior to each EtOH challenge, residents were re-evaluated for baseline FI responding after PO water treatments before each daily session. Three of these trained resident mice lost weight and failed to respond during the day 40 EtOH challenge and were excluded from analyses of these later time points.

Upon completion of *Experiment 3*, blood was collected 10 min after EtOH 1.0 g/kg administration on the last EtOH challenge day from the submandibular vein. Blood samples were centrifuged at 4°C for 10 min at 3,000 rpm, and plasma (5 μL) was extracted for blood ethanol concentration (mg/dL) analysis (AM1 Alcohol Analyzer, Analox Instruments Inc., Lunenburg, MA, United States).

#### **Experiment 4:** Role of iGluRs During the Expression of Alcohol-Escalated Motivation to Fight

After establishing stable rates of FI responding, male residents (*n* = 20) were habituated to an oral administration of tap water via gavage 10 min prior to each daily FI session, as described above. These mice were then given either water (*n =* 10) or EtOH (2.2 g/kg/day, PO, *n* = 10) 10 min prior to their daily FI session for the next 7 days. Ten days later, the role of iGluRs on FI responding and fighting performance was assessed 10 min after an EtOH administration (1.0 g/kg, PO). Specifically, every 72 h these male residents were administered an IP injection of the AMPA receptor antagonist NBQX (0, 10, 17, and 30 mg/kg), the NMDA receptor antagonist ketamine (0, 5.6, 7.5, and 10 mg/kg) or the NMDA receptor antagonist dizocilpine (0.01, 0.1, and 0.3 mg/kg). Each iGluR antagonist dose was administered 15 min before EtOH (1.0 g/kg, PO). Ten minutes after receiving EtOH, FI responding for the opportunity to fight and fighting performance were measured.

#### **Experiment 5:** Effect of Repeated Alcohol Administrations on Locomotor Activity in an Open Field

A separate cohort of mice were habituated to oral administrations of tap water via gavage for 1 week. These mice were given either water (*n =* 8) or EtOH (1.8 or 2.2 g/kg/day, PO, *n* = 7/EtOH treatment) immediately prior to locomotor assessments for the next 7 days. The locomotor behavior of each mouse was observed in a 51 cm× 36 cm × 31 cm plastic enclosure (Rubbermaid) that served as an open field. The total distance traveled (cm) was measured using video tracking software (EthoVision, Noldus, Wageningen, Netherlands). Mouse images were captured under red illumination at a rate of three samples/second through a 0.5-lux camera (Cohu, Model 4815–2100/AL09), which was positioned 165 cm above each open field. Three days after their seventh oral EtOH or water administration, locomotor activity was again assessed for 1 h for three consecutive days (experimental days 10–13) in response to a gavage administration of either water, 1.0 or 2.0 g/kg EtOH, in a semi-randomized order.

### Video Analysis

Agonistic behavior was recorded using a digital webcam (Logitech^®^ HD Pro Webcam C920, Newark, CA, United States). A trained observer (intra-observer reliability: *r* > 0.95) analyzed video recordings during the fixed-interval and the aggressive encounter of the male residents using Observer XT software (Noldus). The first 60 s of the FI, the last 60 s of the FI, and the 60 s aggressive confrontation were analyzed. Key presses on a custom-made keyboard coded the frequency, duration, and latency of each operationally defined behavior (**Table 1**). Aggressive behaviors quantified during social confrontations included attack bites and sideways threat. Non-aggressive behaviors included anogenital and nasal contact, pursuit, self-grooming, rearing, and walking (Miczek and O’Donnell, 1978). Arousal behaviors included tail rattle, digging, and jumping (Krsiak and Steinberg, 1969).

**Table 1:** Effects of repeated oral EtOH (1.8 g/kg, PO) on motor behaviors during fixed interval (FI) responding and aggressive performance during a social confrontation.

	First minute of FI-10				Last minute of FI-10				Resident–Intruder confrontation(1 min)			

	Water	Day1	Day 3	Day 5	Water	Day 1	Day 3	Day 5	Water	Day 1	Day 3	Day 5
Attack latency (s)	–	–	–	–	–	–	–	–	3.10 ± 0.44	**24.7 ± 5.6**	**28.4 ± 6.7**	**41.9 ± 6.7**
Pursuits	–	–	–	–	–	–	–	–	2.70 ± 0.56	3.30 ± 0.90	1.50 ± 0.56	1.79 ± 0.92
Tail rattles	–	–	–	–	–	–	–	–	6.90 ± 0.97	**4.30 ± 1.0**	**2.40 ± 0.55**	**1.90 ± 0.63**
Contacts	–	–	–	–	–	–	–	–	0.32 ± 0.18	**2.40 ± 0.87**	**2.00 ± 0.60**	**2.90 ± 0.66**
Walking (s)	20.0 ± 0.96	19.9 ± 1.1	21.3 ± 0.88	22.3 ± 0.87	13.3 ± 1.0	13.3 ± 1.1	14.4 ± 1.0	22.3 ± 1.3	22.1 ± 0.69	21.6 ± 1.1	22.2 ± 0.51	34.1 ± 2.8
Rearing (s)	9.20 ± 0.95	9.80 ± 1.1	9.80 ± 0.66	9.60 ± 0.69	12.3 ± 1.7	6.30 ± 0.81	6.60 ± 0.54	6.20 ± 0.87	2.50 ± 0.59	3.90 ± 0.75	8.30 ± 0.87	8.60 ± 1.2
Self-groom (s)	3.10 ± 0.63	4.50 ± 0.77	3.50 ± 0.75	3.60 ± 0.29	1.70 ± 0.86	3.80 ± 1.2	0.91 ± 0.52	2.20 ± 1.1	0.09 ± 0.06	0.00 ± 0.0	0.14 ± 0.14	0.02 ± 0.02
Digging (s)	0.92 ± 0.29	0.45 ± 0.18	0.90 ± 0.25	1.00 ± 0.20	2.50 ± 0.61	1.97 ± 0.51	2.60 ± 0.52	3.10 ± 0.66	0.05 ± 0.05	0.00 ± 0.0	0.07 ± 0.06	0.00 ± 0.0
Jumps	1.00 ± 0.63	0.54 ± 0.32	1.40 ± 0.40	3.70 ± 0.93	2.20 ± 0.59	2.80 ± 0.75	7.80 ± 1.7	6.90 ± 1.4	0.01 ± 0.01	0.39 ± 0.22	0.57 ± 0.16	1.20 ± 0.37

*Values that are *bold* indicate significant changes from water administration (*p* < 0.05).*

### Drugs

NBQX, ketamine and dizocilpine were obtained from Tocris Bioscience (Minneapolis, MN, United States). All compounds were dissolved in 0.9% NaCl. Each drug dose was injected intraperitoneally (IP) in a volume of 1 ml/100 g of body weight. For EtOH procedures, 95% ethyl alcohol was purchased from Pharmco-AAPER Products, Inc (Brookfield, CT, United States) and diluted with tap water to obtain 5%, 10%, 18%, or 22% EtOH concentrations (w/v). It was administered via gavage (PO) in a volume of 1 ml/100 g of body weight.

### Statistics

To observe the acute effects of EtOH on motivated responding for aggression reward, time-stamps of each nose-poke during a 10 min FI were carefully examined. All mice were administered either water or EtOH (0.5, 1.0, or 1.8 g/kg, PO) 10 min prior to the start of the FI schedule. The average rate of FI responding over the FI and the number of attack bites following the FI schedule was compared for each gavage treatment using a one-way repeated measures ANOVA. *Post hoc* comparisons for each dose of EtOH to water were made using Dunnett’s test.

Three separate groups of mice trained to respond under the demands of the FI10 schedule were next examined to determine the effects of EtOH dose (0, 1.8, and 2.2 g/kg) on the induction and expression of alcohol-escalated motivation to seek aggression. For this experiment, two-way repeated measures ANOVA were used to assess the impact of EtOH doses (0, 1.8, and 2.2 g/kg) administered over 7 days on the average rate of responding during the FI, and on attack behavior during reward receipt. After a 10-day EtOH free interval, the effect of water or EtOH (1.0 g/kg) on FI responding and attack behavior were again assessed using a one-way ANOVA. Dunnett’s tests were used to make *post hoc* comparisons between water and EtOH treatments for both ANOVA.

To examine how long the effect of alcohol-escalated responding *persists*, the lower dose of alcohol (1.8 g/kg) was again examined (using a *within-subjects* design) on the rate of nose-poke responding during a 10 min FI schedule for an aggressive reward across seven daily administrations. Daily rates of nose-poking during each FI session were compared using a one-way repeated measures ANOVA. In addition, the average Index of Curvature for each daily FI session was also compared over each of the seven daily sessions using a one-way repeated measures ANOVA. Both of these indices of motivated responding during an FI schedule for aggression reinforcement were again assessed at much later time-points (i.e., after increasingly extended EtOH-free intervals) after being challenged with either water or 1.0 g/kg EtOH. Comparisons between water and EtOH on days 13 and 14, 39 and 40, and 59 and 60 (*respectively*) were made using paired *t*-tests for the average of both response rate and the Index of Curvature. In the case of significance, pairwise comparisons of behavioral elements were made using the Holm–Sidak method. In addition, the frequency and duration of behavioral elements collected and scored by a trained observer over 1 min bins at the beginning and end of the FI, and at the start of the aggressive encounter, were compared for water and EtOH on the 1st, 3rd, and 5th daily oral administrations using a one-way repeated measures ANOVA. These same behavioral elements scored during the FI and aggressive encounters during the three later challenge tests (i.e., day 14, 40, and 60) were compared by paired *t*-tests between temporally complimentary water and EtOH (1.0 g/kg) days.

To examine the neuropharmacology of the persistent expression of alcohol-escalated motivation for aggression, iGluR antagonists were administered prior to EtOH (1.0 g/kg) challenges. For this iGluR antagonism study, one-way ANOVA were initially performed on cumulative FI responding and attack bite frequency data, comparing repeatedly water-treated versus repeatedly EtOH-treated groups (i.e., control or repeated EtOH groups) after acute water gavage. There were no significant differences in measures of motivation or aggressive behavior, so a water baseline was calculated from averaging data across control and repeated EtOH groups. Two-way repeated measures ANOVA were conducted on baseline data after water or 1.0 g/kg EtOH and IP vehicle treatment to detect interactions between acute fluid treatment and history of repeated water or 2.2 g/kg EtOH. Additional two-way RM ANOVA were performed to detect interactions between repeated EtOH treatment and doses of MK-801, ketamine or NBQX administered prior to acute 1.0 g/kg EtOH. All pairwise comparisons were made using the Holm–Sidak method.

Finally, three separate groups of mice were examined to determine the effects of EtOH dose (0, 1.8, and 2.2 g/kg) on the induction and expression of locomotor sensitization to EtOH under the same oral administration conditions used above for studies on schedule-controlled aggression. For this experiment, two-way repeated measures ANOVA were used to assess the impact of EtOH doses (0, 1.8, and 2.2 g/kg, PO) administered on days 1, 3, 5, and 7 on the average distance traveled (cm). After a 3-day EtOH free interval, the locomotor response of these mice to EtOH (0, 1.0, or 2.0 g/kg) was again assessed over three consecutive days (one PO dose condition/day). Specifically, locomotor activity (cm traveled) on each challenge day was totaled across 5 min bins and analyzed using a two-way ANOVA (EtOH treatment × minute). Dunnett’s tests were used to make *post hoc* comparisons between EtOH treatment groups (0, 1.8, and 2.2 g/kg) across 5 min time intervals for the first 30 min of each challenge day.

## Results

### EtOH Dose-Dependently Reduced FI Responding for Aggressive Reinforcement With Bi-phasic Effects on Aggressive Behavior

**FIGURE 2:**
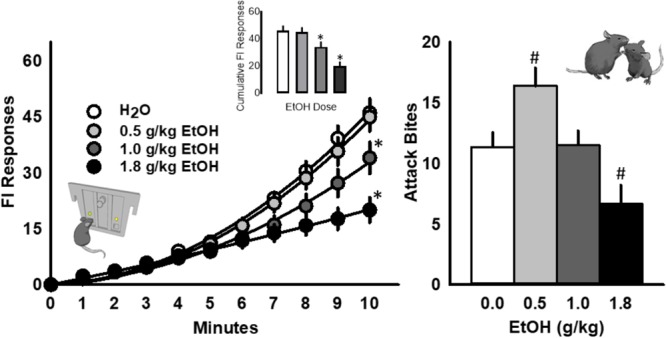
The scallop of operant fixed interval (FI) responding is systematically reduced by acutely administered EtOH (0.5, 1.0, or 1.8 g/kg, PO; **Left**). The frequency of attack bites **(Right)** is significantly increased by 0.5 g/kg EtOH and decreased by 1.8 g/kg EtOH. Significant *post hoc* comparisons to water administration are denoted as ^∗^*p* < 0.05 or ^#^*p* < 0.05.

Acute, oral administration of alcohol dose dependently reduced FI responding for aggression reward [*F*(3, 39) = 14.21, *p* < 0.001; **Figure 2**, left]. The number of attack bites emitted by each resident was increased by EtOH (0.5 g/kg), and decreased by the highest dose (1.8 g/kg) of EtOH [*F*(3, 39) = 21.40, *p* < 0.001; **Figure 2**, right]. Additional behavioral elements, including a longer attack latency [*F*(3, 13) = 4.23, *p* = 0.011], a shorter duration of physical contact [*F*(3, 13) = 7.11, *p* = 0.001] and decrease in tail rattles [*F*(3, 13) = 9.01, *p* = 0.001] were also observed after the administration of the highest dose of EtOH during the aggressive encounter subsequent to FI performance.

### Daily Administrations of Alcohol Dose Dependently Reduced, and Thereafter Escalated, the Motivation to Fight

**FIGURE 3:**
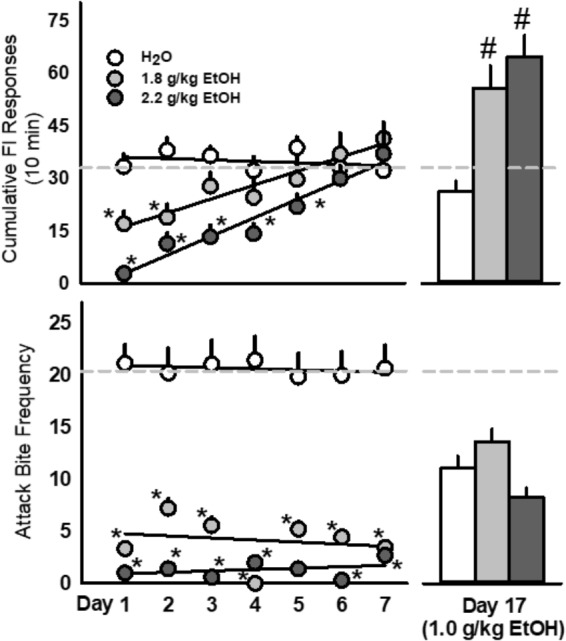
Repeated doses of EtOH (1.8 or 2.2 g/kg, PO; *light gray* or *dark gray*, respectively) produced biphasic effects on aggressive motivation as revealed by rates of fixed interval (FI) responding. Acute EtOH (1.0 g/kg, PO; *light gray*) sensitized FI responding in mice with a history of repeated EtOH exposures **(Top)** whereas fighting performance remained suppressed **(Bottom)**. *Dashed light gray lines* represent the average baseline water values. Significant effects are depicted as: ^∗^*p* < 0.05 compared to water baseline; ^#^*p* < 0.05 compared to the water-treated group.

The rate of FI responding for an aggressive reward was dose dependently attenuated by 1.8 and 2.2 g/kg EtOH over the first few days after administration [*F*(14, 203) = 5.47, *p* < 0.001; **Figure 3**, top]. The amount of aggressive behavior at the completion of each FI was significantly reduced in both groups of EtOH treated mice (1.8 and 2.2 g/kg) following each oral administration [*F*(14, 203) = 4.32, *p* < 0.001; **Figure 3**, bottom]. When challenged 10 days later with EtOH (1.0 g/kg), both groups of EtOH treated mice produced significantly more FI responding for an aggression reward [*F*(2, 29) = 20.29, *p* < 0.001; **Figure 3**], with no notable changes in aggressive performance.

### Repeated Daily Administrations of EtOH Persistently Intensify EtOH-Motivated Responding for an Aggressive Reward

**FIGURE 4:**
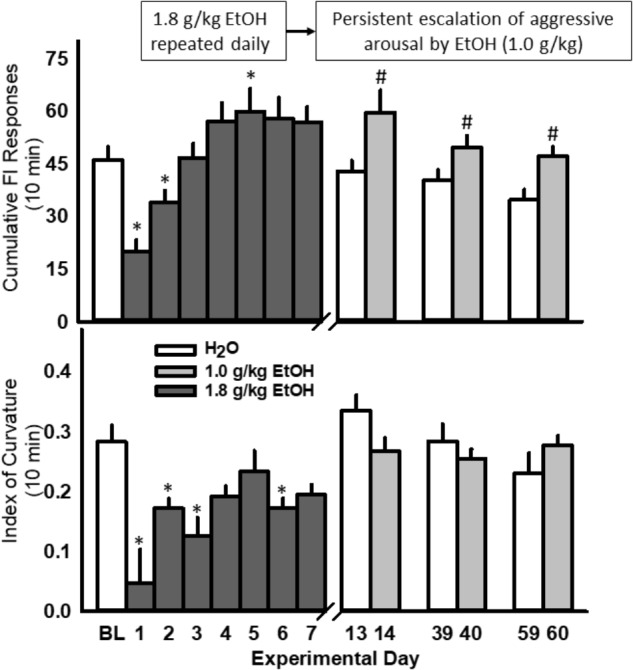
Daily EtOH administrations (1.8 g/kg/day, PO; *dark gray bars*) initially reduce rates of fixed interval (FI) responding within a single cohort of mice **(Top)**; yet, aggressive motivation recovered and exceeded baseline responding by the fifth daily EtOH administration. For more than a month after the final, repeated EtOH administration, an acute EtOH (1.0 g/kg, PO; *light gray bars*) challenge produced a sensitized rate of FI responding. Average daily Indices of Curvature are depicted in **(Bottom)**. Significant effects are depicted as: ^∗^*p* < 0.05 compared to water baseline; ^#^*p* < 0.05 compared to water administration on the previous test day.

The rate of nose-poke responding during a 10 min FI over 7 days was significantly affected by the administration of 1.8 g/kg EtOH within a large cohort of mice [*F*(7, 91) = 18.89, *p* < 0.001; **Figure 4**, top]. Specifically, the first administration of EtOH reduced responding when compared to baseline (water), and this disruptive effect dissipated over the next 3 days, until an increase in motivated responding emerged after the 5th daily administration of EtOH. A sensitization of FI responding for aggression reward was revealed after seven subsequent EtOH free days [day 14, *t*(13) = 3.6, *p* = 0.003], and again on experimental days 40 [*t*(10) = 3.09, *p* = 0.01] and 60 [*t*(10) = 3.5, *p* = 0.005], when all mice were challenged with 1.0 g/kg EtOH as compared to when water was administered the day before (**Figure 4**, top). According to the Index of Curvature, the pattern of FI responding over the course of seven daily EtOH administrations also changed significantly, as responses were found to be more evenly distributed (i.e., IC < 0.3) across the 10 min FI [*F*(7, 85) = 5.02, *p* < 0.001, **Figure 4**, bottom]. Following the FI schedule on days 1, 3, and 5 of EtOH administrations, behavioral elements recorded during the aggressive encounters revealed a longer attack latency [*F*(3, 13) = 15.60, *p* = 0.001], a shorter duration of physical contact [*F*(3, 13) = 6.156, *p* = 0.002] and a decrease in tail rattles directed toward the opponent [*F*(3, 13) = 9.907, *p* = 0.001; **Table 1**]. Interestingly, no differences between EtOH (1.0 g/kg) and water were detected on the amount of motor activation during the FI or on subsequent aggressive behaviors during any of the later challenge days (i.e., see day 14, **Table 2**). Average blood EtOH concentrations were 101.6 ± 5.9 mg/dL 10 min after the last 1.0 g/kg gavage administration.

**Table 2:** A week after repeated EtOH (1.8 g/kg/day, PO) the effects of an acute water or EtOH (1 g/kg) challenge on motor behaviors during fixed interval (FI) responding and aggressive performance.

	First minute of FI-10		Last minute of FI-10		Resident–Intruder confrontation (1 min)	
	Water	EtOH	Water	EtOH	Water	EtOH
Attack latency (s)	–	–	–	–	2.10 ± 0.11	3.60 ± 0.52
Pursuits	–	–	–	–	3.50 ± 0.68	2.00 ± 0.66
Tail rattles	–	–	–	–	5.60 ± 0.74	7.90 ± 0.84
Contacts	–	–	–	–	0.07 ± 0.07	0.32 ± 0.12
Walking (s)	20.1 ± 1.9	16.8 ± 0.70	14.4 ± 0.71	11.8 ± 0.99	16.8 ± 0.81	20.2 ± 0.77
Rearing (s)	12.3 ± 0.86	12.6 ± 0.88	11.0 ± 0.95	13.8 ± 1.2	2.10 ± 0.69	6.00 ± 0.64
Self-groom (s)	2.48 ± 0.18	3.20 ± 0.65	1.70 ± 0.54	1.80 ± 0.89	0.96 ± 0.85	0.35 ± 0.35
Digging (s)	1.54 ± 0.50	1.90 ± 0.46	6.70 ± 1.4	2.80 ± 0.90	0.00 ± 0.0	0.14 ± 0.08
Jumps	5.80 ± 1.9	3.10 ± 0.66	4.90 ± 0.63	3.80 ± 0.65	0.00 ± 0.0	0.29 ± 0.13

### Dizocilpine (MK-801) Recovered FI Responding for Aggression in Controls Given 1.0 g/kg EtOH but Suppressed Aggressive Performance

**FIGURE 5:**
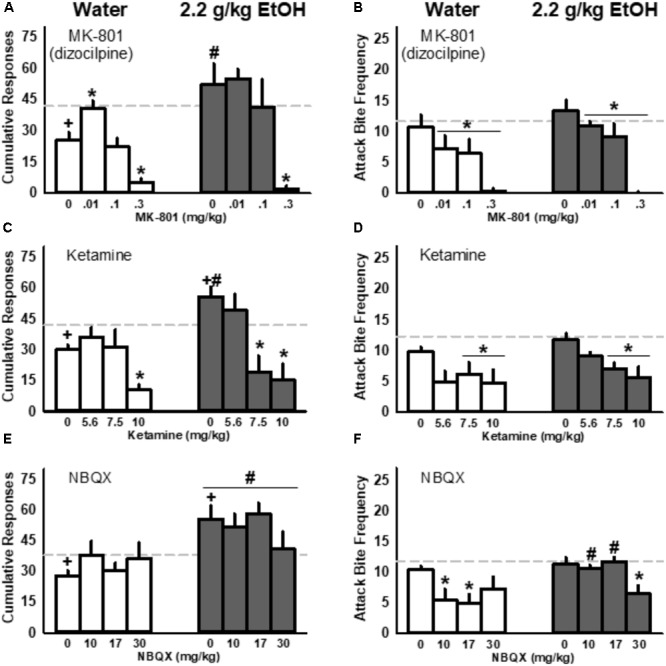
Interactions between ionotropic glutamate receptor antagonists and acutely administered EtOH (1.0 g/kg) in mice repeatedly administered water (*white*) or EtOH (2.2 g/kg, PO; *gray*): responding during the fixed interval (FI; **A,C,E**) and fighting performance **(B,D,F)**. Baseline values in response to water are depicted as *light gray, dashed lines*. Significant *post hoc* comparisons are denoted by: ^+^*p* < 0.05 compared to water baseline; ^∗^*p* < 0.05 compared to vehicle within repeated fluid treatment group (water or 2.2 g/kg EtOH); ^#^*p* < 0.05 compared to water-treated group within that dose.

Acutely administered EtOH (1.0 g/kg) significantly reduced FI responding for aggression reinforcement in control mice with a history of water administrations, while mice with a history of repeated EtOH responded significantly more during the FI than water controls upon receiving the same acute dose of EtOH [*F*(1, 16) = 12.78, *p* = 0.003; **Figure 5A**]. Two-way repeated measures ANOVA also detected a significant interaction between repeated fluid treatment group and acutely administered 1.0 g/kg EtOH and dizocilpine [*F*(3, 48) = 4.13, *p* = 0.011]. Specifically, after receiving 1.0 g/kg EtOH the lowest dose of dizocilpine (0.01 mg/kg) recovered FI responding to baseline in control mice without having any detectable effect on FI responding in mice with a history of repeated 2.2 g/kg EtOH treatments (**Figure 5A**). A main effect of dizocilpine [*F*(3, 48) = 48.82, *p* < 0.001] was driven by suppressed FI responding in both control and EtOH groups treated with 1.0 g/kg EtOH and the highest dose of dizocilpine (0.3 mg/kg). While FI responding for aggression was significantly affected by the historical, repeated administration of water or EtOH, performance during aggressive interactions did not differ between controls and repeatedly EtOH-treated mice that received 1.0 g/kg EtOH. However, two-way RM ANOVA detected a main effect of dizocilpine on attack bite frequency, with all three doses (0.01, 0.1, and 0.3) significantly reducing aggressive behavior [*F*(3, 48) = 29.17, *p* < 0.001; **Figure 5B**].

### Ketamine Suppressed FI Responding for Aggression and Aggressive Performance in Water-Treated Controls and EtOH-Treated Mice Given an Acute Dose of EtOH

Mice with a history of repeated EtOH increased their cumulative FI responding for an aggressive encounter while control mice showed decreased responding upon receiving a 1.0 g/kg EtOH challenge (**Figure 5C**). Two-way RM ANOVA detected this interaction between treatment history (control vs. EtOH) and acute fluid administration [water vs. 1.0 g/kg EtOH; *F*(1, 16) = 12.94, *p* = 0.002] along with a main effect of increased FI responding by historically EtOH-treated mice [*F*(1, 16) = 8.83, *p* = 0.009]. Another two-way RM ANOVA revealed an interaction between EtOH treatment history and ketamine, administered after mice received a 1.0 g/kg EtOH challenge [*F*(3, 48) = 4.79, *p* = 0.005]. EtOH-treated mice responded more during the FI for an aggressive encounter after receiving acute EtOH and the vehicle injection compared to controls. However, these animals were more sensitive to the effects of ketamine and showed a significant reduction in their responding after receiving 7.5 or 10.0 mg/kg ketamine; in contrast, water control animals only reduced their responding when given the highest, 10.0 mg/kg dose of ketamine. Like MK-801, ketamine (7.5 and 10.0 mg/kg) suppressed aggressive performance in both control mice and in historically EtOH-treated animals (**Figure 5D**).

### NBQX Reduced Aggressive Performance Without Affecting Responding for Aggression

Mice given 2.2 g/kg EtOH repeatedly showed an increase in cumulative responding for aggression after receiving an acute dose of 1.0 g/kg EtOH and IP vehicle compared to the water baseline and compared to water-treated controls (**Figure 5E**). In addition to this interaction between repeated EtOH treatment and responding after acute EtOH [*F*(1, 16) = 15.01, *p* = 0.001], an additional two-way RM ANOVA detected a significant main effect of treatment group, indicating increased responding in mice with a history of EtOH treatment compared to water controls when data were collapsed across NBQX dose [*F*(1, 16) = 10.07, *p* = 0.006]. NBQX, unlike MK-801 and ketamine, did not suppress responding for aggression (**Figure 5E**). However, two-way RM ANOVA revealed a significant interaction between EtOH treatment history and aggression after acute EtOH and NBQX [*F*(3, 48) = 4.92, *p* = 0.005], as well as main effects of both historic exposure to EtOH [*F*(1, 16) = 5.47, *p* = 0.033] and of NBQX [*F*(3, 48) = 4.01, *p* = 0.013; **Figure 5F**]. While moderate doses of NBQX (10, 17 mg/kg) diminished aggression in control animals, only the highest dose reduced the number of attack bites inflicted by animals with a history of repeated 2.2 g/kg EtOH treatments.

### Repeated Oral Administrations of EtOH Does Not Engender Locomotor Sensitization to a Later 1.0 g/kg EtOH Challenge

**FIGURE 6:**
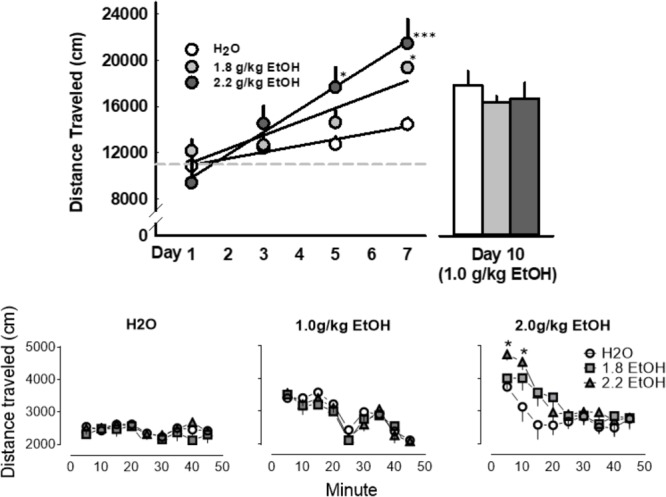
Daily oral administrations of EtOH (1.8 or 2.2 g/kg, PO; *light gray* or *dark gray*, respectively), as compared to water, induced locomotor sensitization as revealed by distance traveled in an open field (**Top, Left**). A later challenge with an EtOH (1.0 g/kg, PO; *light gray*) did not prompt the expression of locomotor sensitization in these mice (**Top, Right; Bottom, Middle**). No conditioned locomotor effects of daily EtOH administrations were apparent when these groups when later challenged with a water gavage (**Bottom, Lef**t). A challenge administration of 2.0 g/kg EtOH, however, did significantly increase locomotor activity in mice that previously received seven oral administrations of 2.2 g/kg EtOH (**Bottom, Right)**. Significant effects are depicted as: ^∗^*p* < 0.05 compared to corresponding water treatment.

Seven daily oral EtOH administrations progressively enhanced locomotor activity, when measured consistently in the same open field, as determined by a main effect for treatment day [*F*(2, 54) = 14.4, *p* < 0.001], and a main effect for treatment group [*F*(2, 54) = 11.1, *p* < 0.001; **Figure 6**, top]. However, in response to a later *water* or *EtOH* (*1.0 g/kg)* challenge in the same context, locomotor activity was not significantly altered between these three groups of mice. In fact, a significant main effect for treatment group [*F*(8, 162) = 9.3, *p* < 0.001] and time-bin [*F*(2, 162) = 7.4, *p* < 0.001] for locomotor activity was only detected during the 2.0 g/kg challenge day. *Post hoc* analyses revealed a selective increase in locomotion within the first 10 min after receiving 2.0 g/kg in those mice previously treated with the highest dose of 2.2 g/kg EtOH (**Figure 6**, bottom).

## Discussion

Appetitive and performance measures in the context of aggression are clearly dissected with the implementation of an FI schedule of reinforcement (Skinner and Morse, 1957). In the present study, FI response curves, with a characteristic scallop shape, were reliably established and stable for more than a month of successive daily sessions. In confirmation of previous observations, aggressive behavior that reinforces FI responding was more intense than species-typical forms of aggression (Fish et al., 2002). A single administration of a low dose of alcohol (i.e., 0.5 g/kg) significantly increased fighting performance, without affecting FI responding. As the dose of alcohol increased, its sedative effect emerged that resulted in the suppression of both behavioral measures. Specifically, alcohol, at both 1.8 and 2.2 g/kg/day, initially disrupted both responding during the FI and subsequent fighting performance. With repeated daily administrations, however, the disruptive effect of these alcohol doses on FI responding quickly recovered to the original response rates, and eventually a *sensitization* of FI responding emerged. The lasting expression of intensified aggressive arousal emerged only when alcohol was administered, such that on days when alcohol was not delivered, both FI responding and fighting behavior were comparable to water-treated control mice. This biphasic action of repeated alcohol on the curvature of FI responding supports previous studies suggesting that this schedule of reinforcement is a sensitive measure of *anticipatory arousal* prior to aggression reward (Skinner and Morse, 1957; Sanger, 1988; Fish et al., 2002).

An acute dose of alcohol (1.0 g/kg) significantly increased FI responding for aggression reward in animals with a history of repeated daily alcohol administrations (1.8 or 2.2 g/kg), whereas the same acute dose of alcohol significantly reduced FI responding for aggression in alcohol-naive mice. The 1.0 g/kg dose of alcohol was selective for FI response rate (i.e., aggressive motivation), and did not differentially affect aggressive performance, or locomotor behavior, in mice with or without a history of receiving daily alcohol treatments (**Table 2** and **Figure 6**). Excitatory and inhibitory amino acid regulatory elements in somatic and terminal regions of the *DA motive system* (Volkow et al., 2017) are promising targets for escalated alcohol drinking and alcohol-heightened aggression (Gourley et al., 2005; Takahashi et al., 2010, 2015; Newman et al., 2012, 2018). Thus, we examined the general role of iGluRs during later challenges with alcohol in mice that were historically treated with alcohol (2.2 g/kg) or water for 7 days.

Under the present conditions, a low dose of the NMDA receptor antagonist dizocilpine (0.01 mg/kg) increased FI responding for aggression in alcohol-naive animals that were treated acutely with 1.0 g/kg alcohol. These same mice significantly reduced their aggressive performance. Because the effects of alcohol and dizocilpine on these behavioral measures were diametrically opposed, it is likely that alcohol suppresses fighting in alcohol-naive animals through a non-glutamatergic mechanism, perhaps by positively modulating GABA_A_ receptor activity (Ticku and Kulkarni, 1988) rather than through a direct and synergistic inhibition of NMDA receptors – the later would be expected to increase fighting (Newman et al., 2018). Unlike dizocilpine, ketamine did not increase FI responding in alcohol-naive mice, which may result from differences in drug kinetics or its interactions with alcohol (Petrakis et al., 2004; Wai et al., 2013).

In contrast with alcohol-naive animals, mice with a history of repeated alcohol exposures were only sensitive to the serenic effects of dizocilpine. Repeated alcohol exposures may upregulate NMDA receptor expression (Haugbol et al., 2005; Wang et al., 2010), thereby preventing dizocilpine from increasing responding in mice that previously received daily alcohol treatments. While the present behavioral findings are suggestive of altered sensitivity to NMDAR antagonists in EtOH-sensitized mice, detailed evaluations of NMDA receptor subtype expression patterns in animals with a history of repeated EtOH exposure and aggression are required to further address this hypothesis. Interestingly, the AMPA receptor antagonist, NBQX, selectively reduced aggressive behavior without affecting FI responding in historically water- or alcohol-treated mice. These data point to a specific role for AMPA receptors in the regulation of schedule-induced aggressive behaviors, but not during the arousal associated with an impending social confrontation. Together, these findings suggest that the behavioral plasticity associated with the long-term expression of escalated motivation for aggression may not be strictly tied to iGluR-dependent mechanisms and are likely to be centered around changes in homeostatic elements of the DA motive circuit. It remains to be determined whether iGluRs are necessary for the induction of this lasting change in behavior, like other types of mesocorticolimbic-dependent behavioral plasticity (Vanderschuren and Kalivas, 2000; Wolf and Ferrario, 2010; Camarini and Pautassi, 2016). It is, however, noteworthy that the current interrogation of iGluRs during schedule-induced “anticipatory” aggression dampens this abnormally intense display of attack behavior unlike the pro-aggressive effects of iGluR antagonists observed specifically in “alcohol-heightened” aggressors, although more detailed analyses of physical confrontations after schedule-induced aggression are required to fully elucidate these findings (Newman et al., 2018).

### Do Fixed Interval Schedules Capture the Motivation to Fight?

The FI schedule of reinforcement effectively separates the behavioral output into appetitive and consummatory components, allowing for the quantification of effort exerted during the interval before having the opportunity to fight and the evaluation of fight performance. Using a 10-min FI schedule results in scalloped response patterns, that can be illustrated mathematically by their index of curvature (Fry et al., 1960). More positive indices of curvature indicate an increasingly greater number of responses generated toward the anticipated end of the interval. In the current study, alcohol increased or decreased the rate of anticipatory FI responses, depending on the frequency of alcohol administrations. While an initial alcohol administration lowered the index of curvature, repeated alcohol administrations induced an alcohol-escalated pattern of FI responding. The “upward shift” in alcohol-escalated FI responding persisted when mice were challenged with a low unit dose of alcohol for more than a month. The selective increase in FI responding, as compared to fighting performance, indicates a specificity toward the motivational aspects of aggressive behavior. Taken together, these results suggest that repeated alcohol exposures in a certain context (i.e., associated with winning a social confrontation) may increase the incentive salience of aggression rewards (Ginsberg and Allee, 1942; Segal et al., 1974; Robinson and Berridge, 1993). While the current study examined oral “gavage” administrations of EtOH, future experimental approaches that allow for self-administered alcohol will provide even more translational value. It will also be interesting to learn if the escalation of FI responding for aggression by EtOH can be generalized to other natural rewards.

Progressive ratio (PR) schedules of reinforcement, from a historical perspective, are more often used to characterize the *motivation* to achieve rewards (Hodos, 1961), including aggression (Golden et al., 2017). PR schedules are considered extinction trials (i.e., the animal ceases to respond at their “breaking point”) and inherently requires multiple reward presentations and consumptions in a single session. Throughout PR schedules, successive reinforcements arguably influence the reward value of each future reinforcement delivery (Stafford et al., 1998). FI schedules measure the acceleration of responding rather than cessation and rely on a single aggression reward per daily session. Employing complex chain schedules of reinforcement may also carefully allow for detailed behavioral analyses of the *motivation* to acquire social rewards like aggression or sex (Everitt, 1990). Nonetheless, the FI schedule used currently was sensitive to both increases and decreases in anticipatory arousal.

### Neural Contributions to the Emergence of Alcohol-Escalated Motivation for Aggression

Alcohol, and its metabolites, directly and indirectly activate VTA DA neurons, ultimately enhancing DA release in terminal areas that are important for reward processing (Di Chiara and Imperato, 1988; van Erp and Miczek, 2007; Plescia et al., 2014; Bassareo et al., 2017). Many DA, and serotonin (5-HT), receptor populations (e.g., DA D1 and D2; 5HT1A, 5HT1B, and 5HT2C) in midbrain, cortical and limbic areas are also critical for the execution of offensive aggression (Nikulina and Kapralova, 1992; Bondar and Kudryavtseva, 2005; de Boer and Koolhaas, 2005; Takahashi et al., 2012). Neural adaptations in response to the repeated actions of alcohol are likely centered around an augmentation of the DA motive circuitry (Volkow et al., 2017). This form of augmented neural plasticity may well be linked to a *sensitization of incentive salience* for the positively reinforcing effects of winning aggressive confrontations (Robinson and Berridge, 1993), and the potential to form an adaptive bias toward hyperexcitability during aggressive *arousal*. Glutamate-dependent forms of plasticity are necessary for the long-term behavioral consequences of alcohol to occur (Brodie, 2002; Broadbent et al., 2003), perhaps altering the perceived outcome of aggression rewards. Indeed, appetitive responses are particularly sensitive to synaptic changes in posterior VTA DA neurons (Wise and McDevitt, 2018), and it is tempting to hypothesize that biogenic amines and amino acids play a concerted role in the development of alcohol-escalated aggressive motivation. In an apparent dissociation from other forms of behavioral sensitization, the *expression* of alcohol-escalated motivation to fight is *not* readily attenuated by iGluR antagonists at dose ranges that are free from disrupting performance measures, as observed herein (**Figure 5**).

Circuits involving anticipatory arousal and the neurobiology of EtOH’s actions also overlap considerably. Hypothalamic and extra-hypothalamic nuclei that are rich in neuropeptides establish such a link (Cannizzaro et al., 2010; Plescia et al., 2014; Pleil et al., 2015; Rinker et al., 2017). It is reasonable to hypothesize that EtOH and its metabolites alter the regulation of sympathetic responses to exacerbate “hot” acts of aggression. Ongoing studies continue to focus on direct and indirect modulation of mesocorticolimbic DA during the expression of alcohol-escalated aggressive motivation. It appears promising to differentiate the neural circuits mediating the urge to fight vs. those responsible for the performance of aggressive acts.

## Author Contributions

HC and KM contributed to designing and conducting the experiments, analyzing the data, interpretation of the results, and writing the manuscript. EN contributed to analyzing the data, interpreting the results, and writing the manuscript. JD and ML assisted with the design of experiments. ST, LW, and WH assisted with experimental procedures.

## Conflict of Interest Statement

The authors declare that the research was conducted in the absence of any commercial or financial relationships that could be construed as a potential conflict of interest.
